# Patients' and Caregivers' Attributes in a Meaningful Care Encounter: Similarities and Notable Differences

**DOI:** 10.5402/2012/320145

**Published:** 2012-06-03

**Authors:** Ingrid Snellman, Christine Gustafsson, Lena-Karin Gustafsson

**Affiliations:** School of Health, Care and Social Welfare, Mälardalen University, P.O. Box 325, 631 05 Eskilstuna, Sweden

## Abstract

In today's healthcare system, there is an imbalance between what patients expect of caregivers' care and their perception of the care they get. How is it possible to reduce this imbalance? The aim of this paper was to describe attributes associated with meaningful encounters in the Swedish healthcare system based on patients' and caregivers' written narratives and to note the differences and similarities between the attributes identified by the two groups. This paper is a qualitative descriptive study. The analysis was guided by qualitative content analyses. Based on patients' narratives, attributes associated with a meaningful encounter fell into four categories: the kind-hearted caregiver, the thoughtful caregiver, the mutually oriented caregiver, and the helpful caregiver. Based on caregivers' narratives, the attributes were categorized as being humane, caring through physical contact, caring by nurturing communication, joy and laughter in care, and a sense of mutuality. The results show that there are both similarities and differences in patients' and caregivers' opinions about the attributes of a meaningful encounter. Knowing more about the attributes associated with meaningful encounters makes it possible for caregivers to individualize care for patients and makes it easier to help and support patients in what they most need support with.

## 1. Introduction

Meeting people in the healthcare system in an acceptable and pleasant way is ascribed great importance these days. A good relationship can be seen as a prerequisite for good and professional care and can have a significant impact on the outcome of patients' care and treatment. This is regardless of the form of care or the categories of staff involved [1, 2].

In today's healthcare system, there is an imbalance between what patients expect of caregivers care and the care that patients perceive they actually get. In what way is it possible to reduce this imbalance? How can encounters in the healthcare system be less superficial and more consistent with patients' expectations so that health and well-being can be promoted? There are several studies which show that patients and families have experienced bad encounters in the healthcare system, but there are few empirical studies of what constitutes experiencing a meaningful encounter.

Empirical research highlights the imbalance between caregivers' and patients' opinions of an encounter's purpose and meaning. In a literature review by Shattell [3], it seems that patients in the care relationship want to be comforted, confirmed, and get to know and become friends with their caregivers. It is also clear that both patients and caregivers are aware that they are in a relationship, but caregivers are unaware of, or perhaps uninterested in patients' experiences and opinions. Caregivers have doubts about the value of the relationship and see no goals or meaning in it. Caregivers feel that they spend a lot of time with patients, but this is not consistent with how patients feel. Another dimension that emerges in Shattell's review was that caregivers exercise power in conflict-ridden care situations, and patients are aware that they are expected to follow a specific agenda. Failure to do so has implications for the individual patient, that is to say, he or she is considered “difficult or hopeless.” Other experiences highlighted in the review are that patients can feel both confirmed and excluded by caregivers. When patients feel left out, they may also feel neglected by those they depend on. Caregivers are seen as “cold” and they “do not have time.” The encounter is regarded as distanced and the patient feels drained, of energy. Contrastingly, in a confirmatory encounter, patients feel that their feelings are taken seriously. Caregivers are perceived as calm and committed, and there is eye contact between the caregiver and the patient. Patients perceive the encounter as energizing [3].

Berg and Danielson [4] emphasize that it is important that patients' resources are used in healthcare and that patients have the opportunity to use their own resources when a caring relationship is created. Adequate information and participation are important for patients when making independent decisions concerning their own health; this is also regulated in Swedish medical legislation [5]. Studies show that older people living in municipal sheltered housing feel excluded from decisions regarding their daily routines, needs, and wishes [6–8]. Older people expect more information and greater opportunities to participate in decision making about their daily care, whereas caregivers believe that they respect patients' wishes and their possibilities to participate in decision making [9]. Hellström and Sarvimäki [10] found that older persons' possibilities for self-determination in sheltered housing were negative. They had neither self-determination nor felt respected and valued as human beings. Their dependency evoked feelings of anger, rage, and submissiveness because of the lack of information and participation in daily routines.

Theoretical research highlights some possible ways of reducing the imbalance between caregivers' and patients' experiences of a good encounter. Snellman [11] suggests the following characteristics: mutuality, equality, acceptance, and confirmation. However, knowing about these characteristics is not enough; the caregiver must also implement them in the care given. In order to make it possible to create such an encounter, it is essential that caregivers are being themselves and do not pretend to be someone else, and they should be personally involved in the care of the patient. Caregivers must also have the ability to create a dialog, to positively influence and support patients in decisions concerning their own lives. Watson's nursing theory [12] highlights that care should be based on a humanistic holistic approach that is both relational and healing. Watson [12, 13] argues that a care relationship refers to values such as deep affection, subjective sense, and shared humanity. Paterson and Zderad's [14] nursing model describes well-being as a goal for caring. Care is seen as a responsible relationship and the caregiver must have the ability to enter into an “I-Thou relationship” with the patient. In the empirical research, there is an obvious imbalance between patients' and caregivers' expectations of care, and there is a lack of empirical research as to what constitutes a meaningful encounter. The aim of this study was to describe the attributes of meaningful encounters in the Swedish healthcare system based on patients' and caregivers' written narratives and further to point out the differences and similarities between the two groups' stated attributes.

## 2. Materials and Methods

### 2.1. Data Collection

A journalist, Catherina Ronsten, collected the data. In 2003, a national advertising campaign was conducted requesting patients, relatives, and healthcare, staff to provide their narratives of meaningful encounters in the Swedish healthcare system. The campaign resulted in 338 narratives. A number of the narratives were selected and worked through. This resulted in an anthology: “Meaningful encounters—people as prescription” [15, 16]. Early in 2011, one of the authors, C. Gustafsson was offered the narratives for research. The narratives were written in the participants' homes, work places, or other places chosen by the participants. The participants' narratives were based on two written questions. The first question was “can you describe an encounter in the healthcare system that you consider as meaningful for you?” Followed by “can you describe your experience of and in that encounter?” The narratives ranged from a half A4 page up to 10 A4 pages.

### 2.2. Participation

A group of researchers was gathered (C. Gustafsson, L.-K. Gustafsson, and I. Snellman), and Ronsten was assigned the task of collecting informed consent from the narrators. Of the 338 persons in total who provided narratives in 2003, 97 were spoken narratives. When asked to write down their narratives, 63 of the 97 had changed their e-mail addresses and could not be contacted. The result was that 275 letters requesting informed consent to use the narratives from the 2003 campaign were sent. Informed consent was received from 128 people (20 relatives, 43 patients, and 65 caregivers) and the narratives were numerically coded and delivered to the researchers. The reasons for nonresponse were informed consent not received because of an unknown address, time constraints when transcribing spoken narratives, or the narrator was deceased.

The inclusion criteria for the present study were written narratives from caregivers and patients; written narratives highlighting some of the characteristics of a meaningful encounter; patients' narratives applied to caregivers and caregivers' narratives applied to patients. Caregivers' narratives were excluded if they focused on animals, relatives, or other caregivers. Patients' narratives were excluded if they focused on something other than caregivers. Some of the narrators contributed more than one narrative. The present study included 33 patients with 63 written narratives and 43 caregivers with 98 written narratives.

### 2.3. Data Analysis

The current paper is a qualitative descriptive study. The analytical process was guided by qualitative content analysis, as described by Granheim and Lundman [17]. Content analysis is an interpretative process, where the context is taken into account [17, 18]. The aim was to describe, without any deeper interpretation, what patients' and caregivers' narratives said about attributes associated with a meaningful encounter and the similarities and differences that exist between caregivers' and patients' opinions. The analysis was performed in several steps (see Table 1). Initially, the narratives were read several times to obtain a comprehensive view of the attributes associated with a meaningful encounter. After that, words and sentences that applied to the attributes which were related by content and context were broken into meaning units by the first author (I. Snellman). Without losing the content of the text, meaning units were condensed and labeled with codes at a low level of abstraction. After rereading and understanding the coded, they were sorted into preliminary categories representing similarities and differences. Finally, the preliminary categories were formulated as categories and subcategories according to the manifest content at a higher level of abstraction. During the analysis process, focus moved between the whole and parts of the text to ensure that interpretations were made at a high level of abstraction. To ensure trustworthiness, the authors discussed and revised borders between categories several times until a unified understanding was reached. To increase the transparency of the interpretation, categories and subcategories are illustrated with quotations. To highlight similarities and differences between caregivers' and patients' opinions as to what attributes they describe as important in a meaningful encounter, the results from both patients and caregivers were analyzed in a similar manner using qualitative content analysis. In the first step, the key findings were collected from patients' and caregiver' results, and the given attributes were listed in a separate file. In step two, the key findings were sorted into two categories based on content from both patients' and caregivers' perspectives. Finally, the differences and similarities were sorted (see Table 4).

### 2.4. Ethical Considerations

Voluntary participation in the study was emphasized. The conducted research was carried out in accordance with the Declaration of Helsinki [19]. Subsequently, all included persons were given verbal and written information and participated after giving informed consent. The narratives were assigned a code number by Ronsten and they were stored in a locked cabinet at Mälardalen University, Sweden. The code number list was stored in another locked cabinet at Ronsten's office, Eskilstuna, Sweden. All participants were guaranteed anonymity towards the researchers.

## 3. Results

The results are presented in three parts. The first part presents the attributes that patients associate with a meaningful encounter, and the second part presents the attributes that caregivers associate with such encounters. The results are summarized in part three, and the differences and similarities between these two groups are presented.

### 3.1. Attributes That Patients Associate with a Meaningful Encounter

Four categories and their underlying subcategories represent the attributes that patients associate with a meaningful encounter (see Table 2). The four categories demonstrate what patients considered important in an encounter with the caregivers. The categories can be seen in two dimensions: what caregivers are and what caregivers do.

#### 3.1.1. The Kind-Hearted Caregiver

This category indicates that a caregiver's personal qualities are valuable in the encounter but also that the caregiver's personal qualities give viability to the patients.

**Caregivers' Valuable Personal Qualities**

Caregivers various personal qualities become visible in patients' narratives. In a frightening situation, a kind, calm, and understanding caregiver is desired. Patients are dependent on caregivers and therefore want to be met by a caregiver who displays humility and empathy. Caregivers with commitment and warmth make a difference to patients in difficult life situations. Patience and sensitivity are other personal qualities that emerged from the patients' narratives. For patients, it is a relief to meet courageous caregivers who are fearless and dare to stay with them when they are in a chaotic health condition experiencing pain or sorrow. *“In the middle of the chaos, there was a person who held our hands, who was fearless in our grief and dared to support us *100%*” *(Narrator 209). Caregivers' courage is also about daring to make demands on patients in a time of sorrow, such as saying goodbye to a loved one.

**Caregivers' Personal Qualities Give Viability**

Meeting caregivers with personal qualities such as kindness, understanding, and courage, as mentioned in the previous section, gives patients hope, and strength and such meetings have healing powers for patients. Patients become more self-confident and dare to trust their caregivers. In these situations, patients are treated as human beings with their own purpose and meaning in life. Patients experience security and love from their caregivers and surrender themselves into their caregivers' hands. When caregivers have and show such personal qualities, patients felt that love emanated from them: *“what I immediately felt, when I met her, was all the love that poured out of her, the calming effect she had … with her help, I live a richer life” *(Narrator 93). The feeling of love comes after a while as patients experience the results of their caregivers' care.

#### 3.1.2. The Thoughtful Caregiver

What caregivers do for patients helps them to deal with their current situations. Two subcategories showing the caregivers' thoughtfulness in what they do for the patients emerged from the analysis: caregivers' care generates calmness and confidence; caregivers' care provides gratitude and physical closeness.

**Caregivers' Care Generates Calmness and Confidence**

Patients reported that their anxiety was reduced if the caregiver created joy in a caring situation, joy also gave confidence. In other caring situations, it was the caregivers' ability to listen to patients' worries that generated confidence and calm. Patients' feelings of calmness also led to improved sleep, and when caregivers admitted shortcomings in front of the patients, it inspired confidence.

**Caregivers' Care Generates Gratitude and Physical Closeness**

When patients experienced responses in caring encounters it made them feel safe and grateful, feelings that they could still experience long after the encounter. *“For the next half hour she is with me, sitting next to me with her arms around my shaking body and helping me gather my scattered soul as though small particles were floating around in the windowless room”* (Narrator 33c). Being present when patients need help, providing physical contact that conveys warmth, and closeness is the care that can help patients feel better.

#### 3.1.3. The Mutually Oriented Caregiver

Being taken seriously and receiving help is something that emerges in the patients' narratives, and the mutuality is demonstrated when both patients and caregivers understand what is happening in the relationships from each other's side. The mutually oriented caregiver emerged in two subcategories: confirmation and mutuality regarding respect and emotional contact.

**Confirmation**

Patients know that they are dependent on their caregivers; therefore, it becomes even more important to be seen and that the caregivers take their patients seriously. *“He did not just see a frail, miserable shell, he looked behind the shell. Saw that there was a man who needed help. He saw me! He made me an offer, if I wanted and dared to, to try and help me feel a little better, to live with my pain”* (Narrator 214). A caregiver who listens and helps in difficult life situations believes, perhaps, that the patient can smile even in life's darkest moments. For example, it means a lot if a caregiver takes the time to sit down at the bedside and confirm the grief that a patient is experiencing.

**Mutuality Regarding Respect and Emotional Contact**

Mutual respect in encounters between patients and caregivers can increase patients' feelings of security. In situations where the caregiver cries with the patient, the tears mean that the patient's situation is such that it touches all people. This mutuality can demonstrate the patient's own power and thus provide an opportunity to grow as a person.

#### 3.1.4. The Helpful Caregiver

The helpful caregiver emerged in the following subcategories: the caregiver provides support and guidance; the caregiver provides time for communication and information.

**The Caregiver Provides Support and Guidance**

Patients appreciated caregivers who gave efficient help which was well thought out and reasonable. Patients appreciated caregivers' inquiring minds. *“But I think of her, I still trusted her judgment. She was both tough and kind. She told it like it was. Even though I did not want to listen”* (Narrator 219). Caregivers' support and assistance give patients the power and strength to move on with their lives. Support can be provided by listening to patients' narratives, but it can also be given by dealing with practical matters such as contacting the funeral director and the social insurance authorities. *“I got all the backing that I needed to cope with the situation we found ourselves in. They helped me grow as a person and to realize what kind of power I have within me”* (Narrator 179). Patients indicated that friendly and rapid assistance is important for their recovery. Being supported in existential questions can give patients the possibility to grow as a person.

**The Caregiver Provides Time for Communication and Information**

For patients, it is important to be given time to talk and when a caregiver facilitates communication with different services within the healthcare system it can make it possible to get through a difficult time in life. It is also important to allow time for relatives to speak with a caregiver as close relatives are often involved in care. From the patients' narratives, it also emerged that knowledge given about their own health status is important as it enables them to cope with their situation. When the caregiver is familiar with the patient's situation and can convey this, it makes a deep impression on the patient.

### 3.2. Attributes That Caregivers Associate with a Meaningful Encounter

Based on caregivers' written narratives, attributes associated with a meaningful encounter with patients are contained in five categories and their underlying subcategories (see Table 3). The five categories paint a picture of what the caregivers consider as important in meaningful encounters with patients. The categories can be seen in two dimensions: the caregivers' human qualities and what the caregivers do for their patients.

#### 3.2.1. Being Human

Two subcategories emerged which highlight “being human” in an encounter with a patient: “being charitable” which highlights personal characteristics the caregiver has, and “showing humanity” which illustrates what is mediated in the encounter when the caregiver is humane.

**Being Charitable**

Being humane is of importance in the encounter with patients and the humane qualities that emerged are being a warm and calm person who brings inspiration and harmony into the patient's life. Tranquility within a caregiver may also be shown when arranging the room of a deceased with dignity. Other human qualities considered as important are being empathetic, open, and present to the patients. Being present and empathetic in the encounter enables caregivers to get to know their patients better. *“In my encounters with people, I will continue to try to find out … *‘*who you are who I will be helping today' *…* and how I can help you”* (Narrator 191). Courage is another personal quality that is found in the narratives, as in daring to suggest and explore new possibilities but also daring to show warmth and physical closeness such as hugging.

**Showing Humanity**

Providing security and showing respect for patients can show humanity. One caregiver wrote: *“then he smiled very weakly, scarcely audibly he said that it smelled ‘me' on my hands and that he felt confident that I was there” *(Narrator 9). Caregivers said that helping patients with their needs and also showing that you care about patients in a sincere manner shows respect. The caregivers sincerely supported patients' participation in their care as much as they have physically could participate, but they also respect the decisions patients make regarding their health, by respecting patients' autonomy. *“The woman was involved in what she could do and did and I felt that this gave her great pleasure and satisfaction”* (Narrator 206). Respect can also be shown for a deceased in a reverent manner, for example, by arranging the room in a dignified manner, making the bed and lighting candles.

#### 3.2.2. Care through Physical Contact

Close physical contact emerged in two subcategories: showing care through physical contact and showing compassion through physical contact.

**Showing Care through Physical Contact**

Through physical contact, caregivers can show their caring attitude. Sitting next to the patient and holding hands, stroking the patient's cheek or arm, shows a caring attitude. *“A hand to hold, a hand that turns the pillow, someone who moistens lips, someone who sees what to do when you are not able or can say it” *(Narrator 134). When caregivers observe patients' needs and feel patients' satisfaction, such as a smile or a thank you, it gives warmth to the caregivers.

**Showing Compassion through Physical Contact**

Through physical contact, caregivers also show love and closeness in their encounters with patients. This is shown by warm hugs. *“I tap gently on the tip of his nose and joke a bit. Then you say, ‘come closer'. I know what it is about, so I put my cheek against yours. You put your arm around my neck and hold tight. Closeness, for a few minutes, it's like that. No one says anything. When I get up, you have fallen asleep” *(Narrator 172b). With physical closeness, caregivers receive signs from patients who show that they recognized and trusted them.

#### 3.2.3. Care by Nurturing Communication

Two subcategories were revealed in this category, demonstrating two means of communication.

**Communication with Words**

Nurturing communication can mean a communion between caregivers and patients. Communication usually occurs when doing something together, such as drinking coffee or watching TV. Communication can be relaxing and empower patients to make their own decisions regarding their health. *“I … sat down next to the little lady and held her hand and began to talk calmly about her condition and which hospital and ward she was in, about myself and the ward and the room she's in and the other patients in the room and who they were and also explained the different sounds that came from different machines in as much detail as possible. The little lady calmed down and talked about herself and seemed completely clear at that time. She ate a sandwich and drank some coffee and seemed very calm. I put the buzzer in her hand and said I'm going to look after some other patients and that she could call whenever she needed. After half an hour she called and when I went in and took her hand and asked what she wanted, she squeezed my hand and placed it on her heart and asked ‘are you an angel'"* (Narrator 182). When caregivers have the ability to create a caring encounter, it is possible for patients to talk about their worries, and caregivers can get a real picture of the patients' situations.

**Communication through Body Language**

Sometimes patients lack the will to talk when they are tired and very sick. In such cases, it may be possible to communicate with a friendly face or gentle hands. Sometimes words are not important, and silence can be a way of communicating with patients, soundless communication. *“She was so tired and sick; she did not say anything in words. But her whole face was one big smile, and she held my gaze all the time, which she had not previously done. It seemed as if she wanted to say, I'm having a good time now and we shall be happy”* (Narrator 216). Through a gaze, communication is established and says more than words. Through eye contact, honesty and directness are created.

#### 3.2.4. Joy and Laughter in Care

Two subcategories emerged from the category: one showing that when caregivers are doing something for a patient, and the act can create joy but that joy can also occur in moments of togetherness.

**Caregivers' Care Gives Pleasure and Satisfaction**

Doing something which gives joy and pleasure to a patient can, in turn, create beneficial feelings of well-being in the caregiver. Creating joy might include doing the patient's hair or dancing with the patient. Just talking kindly to the patient can create happiness. Caregivers can support patients, and this might give them both strength and joy. Humor and jokes sometimes reduce patient stress and anxiety.

**Moments of Joy and Happiness**

Sometimes pleasure is not preceded by an act; it is time together that creates joy and laughter. Moments of humor and laughter may have their place even though the patient is seriously ill.

#### 3.2.5. A Sense of Mutuality

The following three subcategories show different degrees of mutuality: common interests and shared happiness; meeting the patients' needs and confirmation.

**Common Interests and Shared Happiness**

It is important for caregivers to experience shared happiness with patients, as well as common interests and activities such as listening to music. Other shared moments can be about empathizing with the patients, for example, by crying together.

**Ensuring the Patients' Needs Are Met**

The caregivers' narratives revealed that they considered it important to ensure that patients' needs are met. When a patient is not able, the caregiver takes the patient's place and provides the ability. When caregivers take the time to listen to what patients have to say, they are able to satisfy the patients' wishes.

**Confirmation**

In the caregivers' narratives, it emerged that they strive to see each patient and enable patients to develop their health. When caregivers confirm patients' grief and longing, it can sometimes produce calm.

### 3.3. Similarities and Differences between the Attributes Patients and Caregivers Associate with a Meaningful Encounter

The results show that there are both similarities and differences in patients' and caregivers' opinions about the attributes associated with an important encounter. The similarities are that both caregivers and patients believed that attributes such as tranquility, empathy, courage, and warmth are important personal qualities, and that when these qualities are used when caring, the patient is taken seriously, is confirmed, and mutuality is created (see Table 4). The difference between patients' and caregivers' opinions lies mainly in the list of personal qualities in caregivers requested by patients. Patients believe that humility, commitment, kindness, sensitivity, and so forth are important personal qualities. These qualities are not mentioned by the caregivers. On the other hand, caregivers state that physical contact is important, while patients do not mention this. Caregivers also state the importance of being able to use humor to communicate with patients, while patients do not mention this in their narratives (see Table 4).

## 4. Discussion

This study showed both similarities and notable differences between the patients' and the caregivers' opinions on the attributes associated with a meaningful encounter. The results indicated two aspects of the attributes common to both perspectives: one is personal qualities—being kind-hearted and humane. The other aspect is what caregivers do in the encounter, for example, provide support in various situations. The important personal qualities, which both patients and caregivers agree on, are tranquility, empathy, courage and warmth. When caregivers show and use these qualities, patients feel they are being supported, taken seriously and have a sense of mutuality. Caregivers, on the other hand, feel that they can give support, care in a sincere manner, and have the ability to create mutuality with their patients (see Table 4).

The results indicated that both sets of participants agreed on the attributes of respect and mutuality in a caring encounter. A relationship with both respect and mutuality is, according to Tarlier [20], a responsive relationship in the sense that both partners participate in the process. In such a relationship, both are dependent on and reflect on personal morals. The caregivers must demonstrate respect for their patients and the patients, in turn, hold their caregivers in some respect. Mutuality is an important attribute in a meaningful encounter both for patients and caregivers. According to Snellman [11, 21], Paterson and Zderad [14], Mok and Chiu [22], and Buber [23, 24], it is not possible to achieve full mutuality in a care encounter because the caregivers always know more about the patients than the patients know about the caregivers. The relationship is asymmetrical. Another attribute, which both patients and caregivers agreed on, is being confirmed. In both theoretical and empirical research, this is a well-known attribute (see, e.g., [11, 12, 23–26]). To be confirmed, as shown in our results, means being seriously concerned about each other, recognizing a patient's potential and helping him or her to grow as a person.

The results also showed that patients and caregivers have different opinions as to which attributes should be associated with a meaningful encounter. The patients emphasized several important personal qualities that caregivers did not. These included humility, kindness, patience and efficiency. When caregivers demonstrated these qualities, patients felt like they were being treated as human beings and they received information and responses from the caregivers. On the other hand, caregivers considered physical contact, joy and laughter in care and nurturing communication most important. Patients mentioned nothing about this in their narratives. Studies performed by Shattell [3] and Berg and Danielson [4] have shown an imbalance between caregivers' and patients' opinions concerning the care encounter's purpose and meaning. Both were aware that they were in a relationship, but they had different expectations. It can be difficult for the patient to use his or her capabilities if the caregiver has totally different expectations. Relationships between patients and caregivers are asymmetric and, because of this, patients are always more vulnerable and have less power than the caregivers. According to Oudshoorn et al. [27], one consequence of this imbalance in the care relationship, can be demonstrated through caregivers' exercise of power. An example of this is the dominance gained by having information about the patients that the patients did not choose to disclose. Caregivers have the power to go to family members to find out what they want to know.

The caregivers pointed to physical contact and humor in care as important attributes in a meaningful encounter. According to Mallett and A'Hern [28], the importance of humor is to facilitate communication between caregivers and patients and to ensure sensitivity and understanding when to provide care. This is, of course, very important but when patients spoke of their experiences they said nothing about physical contact or humor. This is an important difference to be aware of because both physical contact and humor in care can violate patients' integrity and privacy [29]. Patently, there will be times when humor is not appropriate [30]. However, according to most studies [31–33] humor in care is something valuable which fosters a relationship, eases tension and more. Using physical contact and humor when caring for a patient requires great mindfulness. It is a balancing act. According to Routasalo's review [34], how patients experience physical contact is always individual and sensitive to external factors. The review also showed that the effects of contact by holding hands were interpreted as positive in 70% of cases and interpreted as negative in 30% of cases. An expressive caress of the face caused discomfort and contact was experienced as negative when it involved some intimate procedures or when it was not consistent with the patient's needs. The result of this study indicated that caregivers must be careful when they use physical contact in caring and be observant when patients do not want to be physically touched.

## 5. Conclusions and Implications in Practice

The study's results highlight both similarities and differences when caregivers and patients express which attributes are important to them in a meaningful encounter. The similarities in opinions show that patients and caregivers agree on some personal qualities for caregivers but also that patients felt supported when caregivers used their personal qualities. The results also highlight patients' and caregivers' different opinions on the attributes associated with a meaningful encounter. These differences do not necessarily lead to poor care but it is very possible that they could. Identifying common attributes in the results provides caregivers with important knowledge and makes individualized care of patients possible.

The caregivers' awareness of these attributes is important in patients' care so that the imbalance between expectations and perceived care can be decreased. In care, it is important that a meaningful encounter can be created as often as possible and therefore agreeing on expectations is necessary. A sense of confidence is a prerequisite if patients want to talk about something in their life situation. When caregivers know something about patients' wishes and desires, it is possible to create a meaningful encounter with attributes which both partners agree on. It is the patient's story about herself that enables a nuanced and meaningful encounter. Being aware of what attributes the patients experience is important and helps caregivers to individualize care for the patient. It makes it easier to help and support the patient with what he or she most needs support with. We also think that caregivers need to develop their ability to create a good caring encounter in nursing training. One suggestion is that nursing students can develop using these common attributes in a meaningful encounter in a caring situation. For example, in educational drama students can practice using personal qualities such as tranquility, empathy, courage, and warmth within a caring situation so that patients feel that they are being supported, taken seriously and can meet the caregiver in a mutual way.

##  Conflict of Interests

The authors declare that they have no conflict of interests with respect to the authorship and/or publication of this paper.

##  Funding

This paper was supported by Mälardalen University, Sweden and Rekarne Sparbanksstiftelse, Sweden.

## Figures and Tables

**Table 1:** Examples of meaning units, condensed meaning units, codes, subcategories, and categories.

Meaning unit	Condensed meaning units	Code	Subcategories	Categories
Otherwise kindness and interest among caregivers was great towards patients. Kindness, we put great value on. (Narrator 12)	Kindness is of great value	Kindness	Caregivers' valuable personal qualities	
With her courage to meet my pain, she gathers, piece by piece together my shattered soul. (Narrator 188)	With her courage to meet my pain, she gathered my soul	Courage		The kind-hearted caregiver
Her calmness and caring had a strong influence on me. (Narrator 200)	Her calmness makes a deep impression	Calm	Caregivers' personal qualities gives viability	

**Table 2:** Attributes that patients associated with meaningful encounters with caregivers.

Subcategories	Categories
Caregivers' valuable personal qualities Caregivers' personal qualities give viability	The kind-hearted caregiver
Caregivers' care generates calmness and confidence Caregivers' care generates gratitude and physical closeness	The thoughtful caregiver
Confirmation Mutuality regarding respect and emotional contact	The mutually-oriented caregiver
The caregiver provides support and guidance The caregiver provides time for communication and information	The helpful caregiver

**Table 3:** Caregivers given attributes of meaningful encounters with patients.

Subcategories	Categories
Being charitable Showing humanity	Being human
Showing care through physical contact Showing compassion through physical contact	Care through physical contact
Communication with words Communication through body language	Care by nurturing communication
Caregivers' care gives pleasure and satisfaction Moments of joy and happiness	Joy and laughter in care
Common interests and shared happiness Ensuring the patient's needs are met Confirmation	A sense of mutuality

**Table 4:** Similarities and differences between patients and caregivers narratives about a meaningful encounter.

Patients' perspectives		Patients' and caregivers' perspectives	Caregivers' perspectives	
Caregivers' personal qualities	Caregivers' shown/used personal qualities		Caregivers' personal qualities	Caregivers' shown/used personal qualities
		Similarities		

Tranquility	Give support/assistance	⇔	Tranquility	Give support
Empathetic	Taken seriously		Empathetic	Show that you care in a sincere manner
Courage	Be confirmed		Courage	Be confirmed
Warmth	Create mutuality		Warmth	Create mutuality
	Granted time			Give time
	Create joy			Create joy and pleasure

		Differences		

Humility	Give response	⇔	Openness	Joking
Commitment	Always be there		Be present	See what patients need
Patience	Guiding			Physical touch
Sensitive	Give information/knowledge			Eye contact
Efficient	Patients treated as persons			Create moment of togetherness
Inquiring mind	Ability to listen			Replace patients ability
Kindness	Admit shortcomings			Give opportunities
Understanding				Show respect
				Helping patients with what they want help with
				Nourishing communication (i) with words (ii) without words Talk kindly
